# Boron neutron capture therapy for vulvar melanoma and genital extramammary Paget’s disease with curative responses

**DOI:** 10.1186/s40880-018-0297-9

**Published:** 2018-06-19

**Authors:** Junichi Hiratsuka, Nobuhiko Kamitani, Ryo Tanaka, Eisaku Yoden, Ryuji Tokiya, Minoru Suzuki, Rolf F. Barth, Koji Ono

**Affiliations:** 1 Department of Radiation Oncology, Kawasaki Medical School, 577, Matsushima, Kurashiki, Okayama 701-0192 Japan; 2 Department of Dermatology, Kawasaki Medical School, Kurashiki, Okayama 701-0192 Japan; 3 Particle Radiation Oncology, Kyoto University Research Reactor Institute, Osaka, 590-0494 Japan; 4 Department of Pathology, The Ohio State University, Columbus, OH 43210 USA

**Keywords:** Boron neutron capture therapy, Vulvar melanoma, Extramammary Paget’s disease, Penis, Vulva

## Abstract

**Background:**

Although the most commonly recommended treatment for melanoma and extramammary Paget’s disease (EMPD) of the genital region is wide surgical excision of the lesion, the procedure is highly invasive and can lead to functional and sexual problems. Alternative treatments have been used for local control when wide local excision was not feasible. Here, we describe four patients with genital malignancies who were treated with boron neutron capture therapy (BNCT).

**Methods:**

The four patients included one patient with vulvar melanoma (VM) and three with genital EMPD. They underwent BNCT at the Kyoto University Research Reactor between 2005 and 2014 using para-boronophenylalanine as the boron delivery agent. They were irradiated with an epithermal neutron beam between the curative tumor dose and the tolerable skin/mucosal doses.

**Results:**

All patients showed similar tumor and normal tissue responses following BNCT and achieved complete responses within 6 months. The most severe normal tissue response was moderate skin erosion during the first 2 months, which diminished gradually thereafter. Dysuria or contact pain persisted for 2 months and resolved completely by 4 months.

**Conclusions:**

Treating VM and EMPD with BNCT resulted in complete local tumor control. Based on our clinical experience, we conclude that BNCT is a promising treatment for primary VM and EMPD of the genital region.

*Trial registration* numbers UMIN000005124

## Background

Although vulvar cancer is generally considered rare, it is the fourth most common gynecologic malignancy in the United States [1]. Vulvar melanoma (VM) is the second-most common type of cancer involving the vulva after squamous cell carcinoma and usually occurs in the fifth to seventh decades of life. VM accounts for 5%–10% of vulvar cancers and has an incidence of 0.2 per 100,000 women in the United States [2]. Mert et al. [3] have reported differences in clinicopathologic features and survival patterns between patients with vulvar/vaginal melanomas and those with cutaneous melanomas from the data of Surveillance Epidemiology and End Results (SEER). Included in the study were 762 patients with vulvar/vaginal melanomas and 55,485 patients with cutaneous melanoma. Twenty-eight patients of the vulvar/vaginal group and 334 patients of the cutaneous group were black (3.6% vs 0.6%, respectively). The median age at the time of diagnosis was 68 years in the vulvar/vaginal group and 52 years in the cutaneous group. Three hundred fifty patients (45.9%) in the vulvar/vaginal and 46,499 patients (83.8%) in the cutaneous group presented with localized disease. The median survival of the black patients was 16 months in the vulvar/vaginal group and 124 months in the cutaneous melanoma group. The median survival in the nonblack population was 39 months in the vulvar/vaginal group compared to 319 months in the cutaneous melanoma group. This study indicated significant differences in median age at diagnosis, racial distribution, and survival of women with vulvar/vaginal melanomas compared to those with cutaneous tumors.

Extramammary Paget’s disease (EMPD) is a rare, slow-growing cutaneous adenocarcinoma of apocrine gland-bearing skin. It presents as an erythematous, eczematous, hyperkeratotic plaque with occasional areas of hypopigmentation and superficial erosions. Histologic diagnosis of both mammary Paget’s disease and EMPD is based on the presence of large infiltrating, round intraepithelial malignant cells with glandular differentiation, which are distributed as individual cells or in clusters [4]. The most common sites of involvement are the vulva in women and scrotal and penile skin in men, and perineal and perianal areas in both men and women. EMPD of the genital region constitutes 1%–5% of all vulvar malignancies in woman, with a peak incidence at age 65 [4]. The incidence of these malignancies in populous Asian countries, such as China, India, and Japan, is unknown to our knowledge, but it is seen mostly in males in Western countries [5].

The most commonly recommended treatment for melanoma and EMPD of the genital region, including the vulva, penis, scrotum, and perianal area, is wide surgical excision of the lesion, with or without lymph node dissection, and reconstruction with a skin graft or a skin flap [6–8]. More recently neoadjuvant chemotherapy has been administered [9]. Although wide surgical excision has been the standard procedure, it is highly invasive, especially in older patients, and it can lead to a variety of functional and sexual problems that result in a poor quality of life [10, 11]. Alternative treatment modalities, such as topical chemotherapy [12], immunotherapy, carbon ion radiotherapy [13], and photodynamic therapy, have been administered for local control when wide local excision was not feasible. Fukuda and Funakoshi [14] recently reviewed current therapies for EMPD and concluded that current systemic chemotherapeutic regimens are not very effective. However, recent genomic analysis indicate increased frequency of mismatch repair mutations in patients with EMPD suggesting that these patients might be candidates for immunotherapy with anti-PD1 antibody [14]. The treatment of vulvar melanomas has been similarly unsatisfactory [15], making a strong case for new therapeutic approaches.

**Fig. 1:**
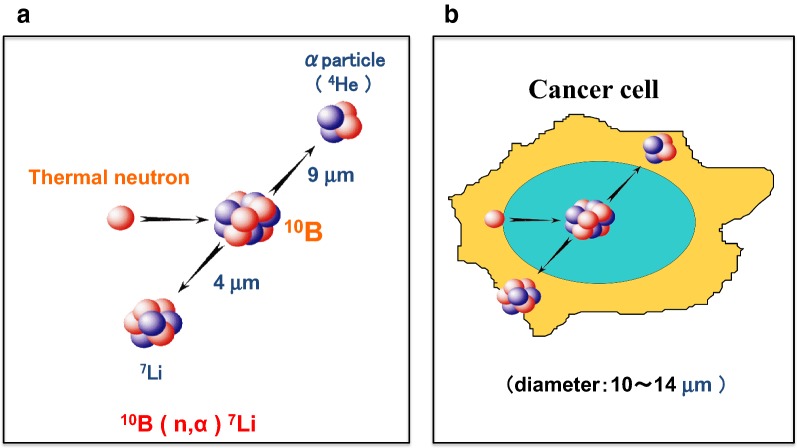
Boron neutron capture therapy. BNCT is based on the nuclear capture and fission reactions that occur when boron-10, a non-radioactive stable isotope, is irradiated with low-energy thermal neutrons or, alternatively, higher-energy epithermal neutrons, which become thermalized as they penetrate tissues. **a** The ^10^B(n,α)^7^Li capture reaction results in the production of high linear energy transfer (LET) alpha particles (stripped down ^4^He nuclei) and recoiling lithium-7 (^7^Li) atoms. **b** A sufficient amount of ^10^B must be delivered selectively to the tumor and, depending upon the depth of the tumor, this can range from ~ 20 to 50 µg/g (~ 10^9^ atoms/cell); a sufficient number of neutrons must be absorbed by the cancer cell to sustain a lethal ^10^B(n,α)^7^Li capture reaction. Since the destructive effects of the α particles are limited to boron-containing cells, BNCT can selectively kill malignant cells and spare surrounding normal cells

Boron neutron capture therapy (BNCT) is based on a nuclear reaction between the non-radioactive isotope boron-10 (^10^B) and either low energy thermal neutrons or higher energy epithermal neutrons (Fig. 1). These are captured by ^10^B atoms, resulting in the production of alpha (a) particles (^4^He) and lithium atoms (^7^Li) (Fig. 1a). The α particles have high linear energy transfer (LET) and very short path lengths (≤ 10 µm), which are approximately the diameter of a single tumor cell (Fig. 1b). If sufficient amounts of ^10^B atoms are selectively localized in tumor cells, the resulting ^10^B(*n*,*α*)^7^Li capture reaction can kill them and spare surrounding normal cells. Theoretically, BNCT is an ideal type of radiation therapy because it is both biologically and physically targeted and the structure and function of normal tissues are spared.

In 1972, experimental studies on BNCT for cutaneous melanoma were initiated by Yutaka Mishima at Kobe University in Japan, and his colleagues, who included physicists, chemists, radiation biologists, and physicians [16, 17]. In 1987, after 15 years of basic research, his team initiated the first clinical use of BNCT to treat a patient with a metastatic melanoma to the skin of the left occipital region of the scalp, using para-boronophenylalanine (BPA) as the boron delivery agent [18]. This was administered by perilesional injections of BPA-HCl, followed by thermal neutron irradiation, which resulted in complete regression of the tumor. This prompted Mishima et al. [19] to treat a patient with an acral melanoma on the sole of the right foot. BPA-fructose (BPA-F), which increased the water solubility of BPA [20], was injected perilesionally, followed by neutron irradiation. Again, there was complete regression of the tumor. Based on the Japanese clinical results, other reports followed that described the treatment of cutaneous melanomas using BNCT [21, 22].

Although BPA was developed as a boron delivery agent for BNCT of melanoma, it selectively accumulates in other types of malignant tumors. Coderre et al. [23] first reported that BPA was taken up by non-pigmented tumors, including a rat brain tumor, the 9 L gliosarcoma. This suggested that other uptake mechanisms existed that are independent of melanin synthesis, which might explain its tumor-localizing properties. The selective uptake of BPA most likely is due to increased l-type amino acid transport activity in tumor cells [24]. Shortly after the report of Coderre et al. BPA started to be used as a boron delivery agent for patients with brain tumors [25] and head and neck cancers [26]. It also has been evaluated for potential use in treating patients with mesotheliomas [27] and colon cancer metastatic to the liver [28]. Here, we report the results obtained using BNCT to treat one patient with VM and three with EMPD.

To our knowledge, the present report is the first describing the treatment of patients with VM and EMPD using BNCT.

## Methods

### Patients

Patient information and tumor characteristics are summarized in Table 1. All of the patients had been referred to the Department of Radiation Oncology, Kawasaki Medical School, to receive BNCT as an alternative treatment because they had refused wide surgical excision. One patient was a 73-year-old woman with VM and three patients, two men and one woman, had EMPD of the genital region. They ranged in age from 69 to 75 years at the time of treatment. Their malignancies were located in the vulva, scrotum, perianal region, and penis, respectively. They received BNCT between November 2005 and April 2014, and all diagnoses were confirmed histologically. The tumors were evaluated by computed tomographic (CT) scans, magnetic resonance imaging (MRI), and visual inspection or palpation immediately prior to BNCT. None of the patients had evidence of regional lymph node involvement, distant metastases, or second malignancies at the time that BNCT was administered. Their Karnofsky Performance scores were all > 70. BNCT was the first-line therapy in three of these patients, and one (Case 1) had received immunotherapy as first-line therapy. They all gave informed consent to undergo BNCT, and approval for this was obtained from the Kawasaki Medical School and the Kyoto University Medical and Ethics Committee.

**Table 1:** Patient and tumor characteristics

Case	Age/gender	Tumor site	Histology	Prior therapy	Tumor diameter	Tumor status	Tumor stage
1	73/F	Vulva	Lentiginous mucosal melanoma	INF-α + chemotherapy	2.5 cm × 4.5 cm	Nodular	T4N0M0
2	75/M	Scrotum to penis	EMPD	None	3 cm × 8 cm	Invasive	T1N0M0
3	73/M	Scrotum to perianal	EMPD	None	5.5 cm × 6.5 cm	Microinvasive	T1N0M0
4	69/F	Vulva to labia	EMPD	None	3 cm × 6 cm	Invasive	T1N0M0

*EMPD* extramammary Paget’s disease, *INF* interferon

### Treatment protocol

All patients were treated according to the treatment procedure developed by Mishima et al. [19, 29]. BNCT was carried out at the Kyoto University Research Reactor (KUR) operating at 5 MW of power using an epithermal neutron beam. In all patients, a 10-mm-thick plate, made of human body equivalent material, was placed over the area to be irradiated to increase the thermal neutron dose delivered to these superficial tumors. The regimen described below for administering BNCT was developed based on previously reported radiobiological factors for dose optimization and boron concentration kinetics [30].

^10^B-enriched L-BPA, purchased from Interpharma Praha (Prague, Czech Republic), was used as the boron delivery agent. BPA-F, which is more soluble in water than hydrochloride [20], was administered by an intravenous drip infusion at a dose of 200 mg/kg body weight over 3 h, at a rate of 80 mg/kg/h for the first 2 h, and at a lower rate of 40 mg/kg/h for the last hour. Neutron irradiation was carried out during the last hour during infusion of BPA-F.

Gold wires and small thermoluminescence detectors (TLD) of magnesium ortho-silicate (Mg_2_SiO_4_) were used to measure neutron flux and γ-ray dose, respectively, and these were attached to the skin or mucosa at the radiation field for dosimetry. Lithium fluoride (LiF) sheets (10 mm thick) were chosen as collimators to shield normal tissues from neutron irradiation. The radiation field included a 3–4 cm safety margin surrounding the visible lesions.

Venous blood was drawn just prior to neutron irradiation; blood boron concentrations were determined immediately by prompt gamma-ray analysis [31]. Skin and tumor boron concentrations were based on data from Fukuda et al. [30], and were determined by multiplying the blood boron concentration by 1.2 and 2.5–3.0, respectively. Boron concentrations of blood, tumor and skin/mucosa of each patient are shown in Table 2.

**Table 2:** Boron concentrations in blood, tumor and skin/mucosa of each patient

Case number	Boron concentration (ppm)		
	Blood^a^	Tumor^b^	Skin/mucosa^b^
1	15.0	45.0	18.0
2	18.0	48.6	21.6
3	11.0	27.5	13.2
4	10.0	25.0	12.0

^a^Determined by prompt gamma activation analysis [31]

^b^Calculated by multiplying blood boron concentration by tumor/blood or normal tissue/blood ratio

BNCT consists of mixed radiation fields that differ in their linear energy transfer (LET). The total radiation dose in Gy, delivered to any tissue, can be expressed in Gray-equivalent (Gy-Eq) units as the sum of each of the high LET dose components multiplied by radiobiological effectiveness (RBE) factors, and more specifically, the compound biological effectiveness (CBE) factors [32]. All absorbed doses were expressed in Gy-Eq units, using these factors.

The minimum dose for tumor control in a single fraction was assumed to be 20 Gy-Eq for EMPD and 25 Gy-Eq for VM. The maximum tolerated doses to the skin and mucosa in a single treatment were assumed to be 18 and 16 Gy-Eq, respectively. A radiation dose that was less than the maximum tolerated dose and greater than the curative dose was selected using the Monte Carlo software package SERA for dose planning [33]. All patients received BNCT without anesthesia.

### Evaluation of local response and survival

Tumor responses were graded as follows: complete regression (CR), complete disappearance and regression of pigment plaque and tumor by visual inspection, CT or MRI; and non-CR, no regression or incomplete regression of plaque and tumor. Complications of normal skin/mucosa and pain were graded according to the Common Terminology Criteria for Adverse Events, v.4.0. We evaluated the local response every 3 months after the therapy. A survival analysis was made in October 2017.

## Results

Tumor responses and complications are summarized below and in Table 3. All lesions regressed completely with depigmentation within 6 months. No local recurrences in the radiation field were observed during follow-up, which ranged from 1.1 to 6.9 years.

**Table 3:** Radiation parameters, tumor responses and complications

Case	Date at BNCT	Irradiation period (min)	Minimum tumor dose (Gy-Eq)	Maximum skin/mucosa dose (Gy-Eq)	Tumor response	Complications	Loco-regional control and clinical outcome
1	2005.11.9	49	29	8.0	CR	Pain (Grade 2)	CR: 1.1 years (death with pulmonary metastases)
2	2010.11.26	65	23	8.7	CR	Erosion (Grade 2)	CR: 6.9 years (alive with NED)
3	2011.2.10	78	18	7.3	CR	Erosion (Grade 2)	CR: 6.5 years (alive with NED)
4	2014.4.17	50	20	6.4	CR	Mucositis (Grade 1)	CR: 3.2 years (death due to heart disease)

*BNCT* boron neutron capture therapy, *CR* complete response, *NED* no evidence of disease

### VM (Case 1)

**Fig. 2:**
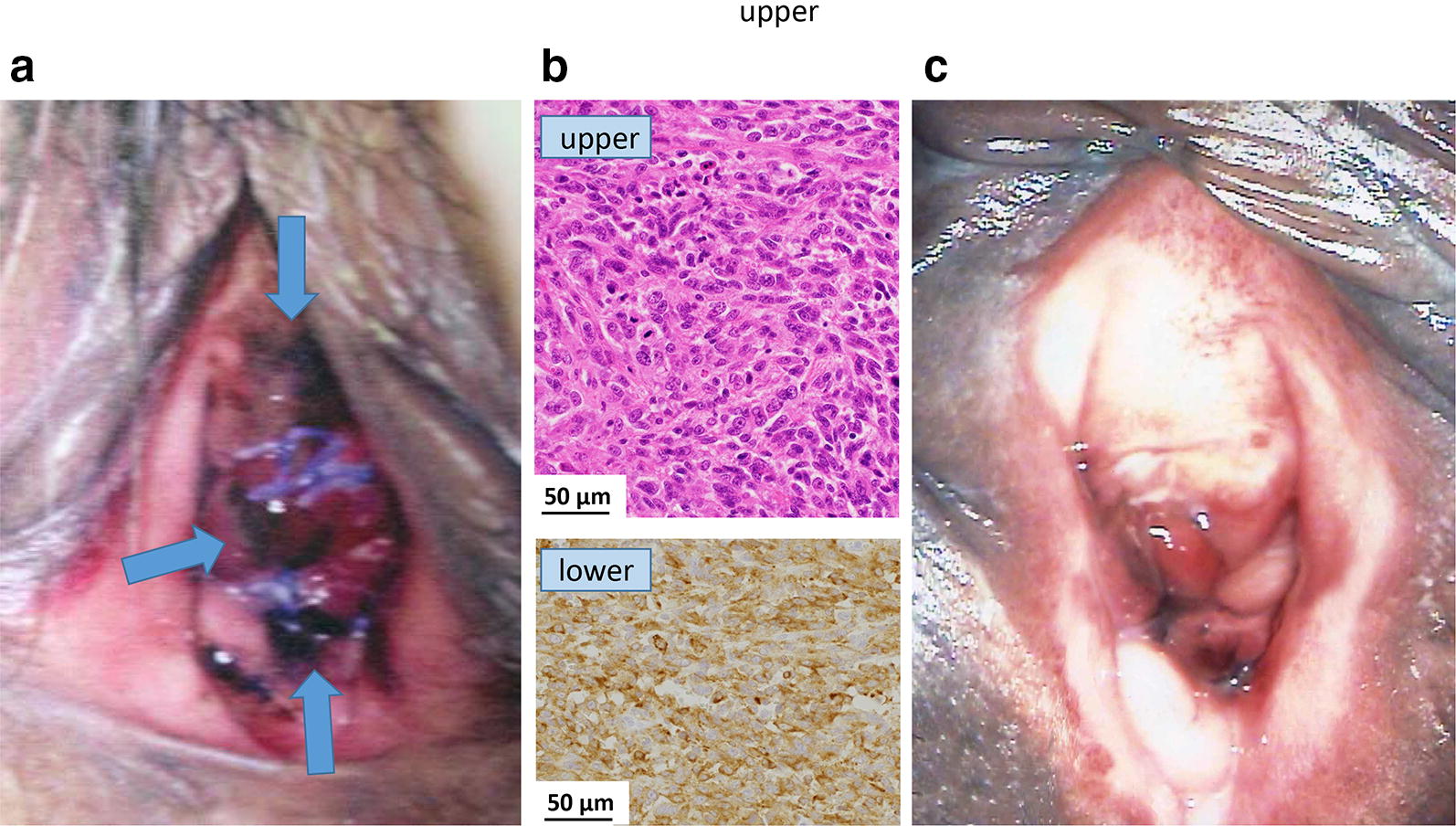
Macroscopic and microscopic images of a 73-year-old woman with vulvar melanoma. **a** External appearance before boron neutron capture therapy (BNCT): a black macula (arrows) on her vulva was asymmetrical in shape and variable in color. **b** Microscopic findings: the dermis showed a massive infiltrate of tumor cells. The cells had large hyperchromatic and irregularly shaped nuclei with multiple mitoses (upper). A small number of pigment cells were seen. Tumor cells were positive for HMB-45 (lower) and S-100. **c** External appearance after BNCT: the absorbed doses were 8.0 Gy-Eq to the normal vaginal mucosa and 29 Gy-Eq to the melanoma. The black macule slowly faded and was no longer visible 4 months later. No severe adverse local side effects such as ulceration nor local recurrence were seen at the site of irradiation. However, she died of disseminated metastatic disease 1.1 years later

A 73-year-old woman presented with a black macule on her vulva (Fig. 2a). A small nodular lesion (1.5 cm) was resected for histopathological examination at the referring hospital and was diagnosed as a lentiginous mucosal melanoma (Fig. 2b). At the time of BNCT, the 2.5 × 4.5 cm flat lesion was asymmetrical in shape and variable in color and had not invaded the vaginal mucosa. There was no evidence of brain, chest, or abdominal metastases. The irradiation time was 49 min. Epithermal neutrons were administered at a maximum dose of 8.0 Gy-Eq to the normal mucosa and at a minimum dose of 29 Gy-Eq to the melanoma. Subsequently, the patient developed slight vulvar swelling and pain after irradiation, but these symptoms resolved almost completely within 1 month. The black macule slowly faded and was no longer visible 4 months later. There were no severe local adverse events such as ulceration (Fig. 2c). Although the patient died of disseminated melanoma 1.1 years later, there was no local recurrence.

### EMPD (Cases 2, 3 and 4)

**Fig. 3:**
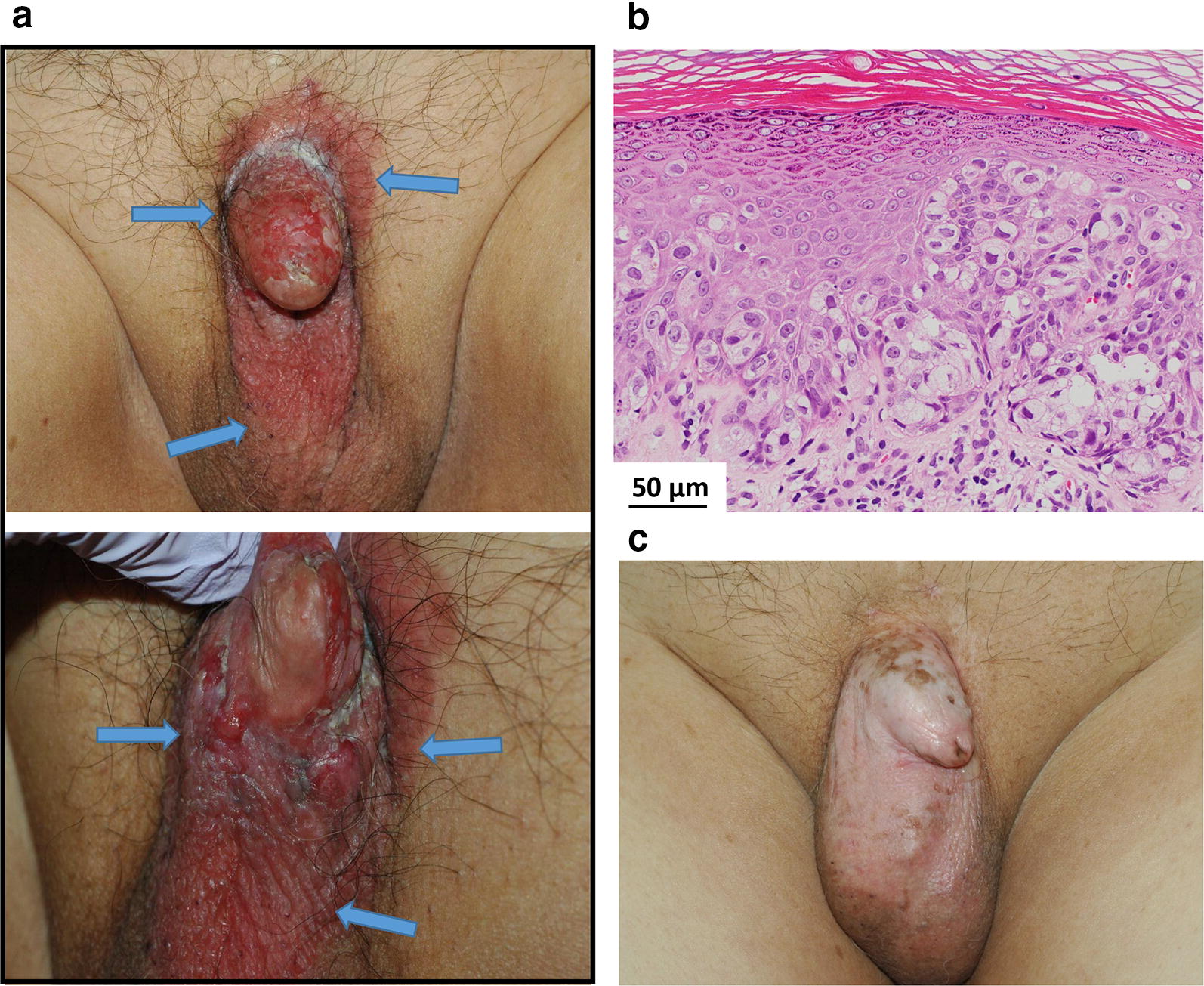
Macroscopic and pathologic images of a 75-year-old man with extramammary Paget’s disease (EMPD). **a** External appearance before boron neutron capture therapy (BNCT): a pruritic, painful and persistent erythematous lesion (arrows) was on the penis and scrotum. Penile atrophy was due to long-term hormonal administration for prostate cancer. **b** Microscopic findings: histopathology was diagnostic for EMPD, as evidenced by large, round, vacuolated, infiltrating intraepithelial malignant cells. The basal membrane was partially unclear. **c** External appearance after BNCT: BNCT delivered an absorbed dose of 8.7 Gy-Eq to the normal skin and 23 Gy-Eq to the tumor. He had a complete response with depigmentation of the lesion, and no severe adverse event (such as ulceration), and was alive and well without evidence of recurrence or adverse effects at 6.9 years after BNCT

**Fig. 4:**
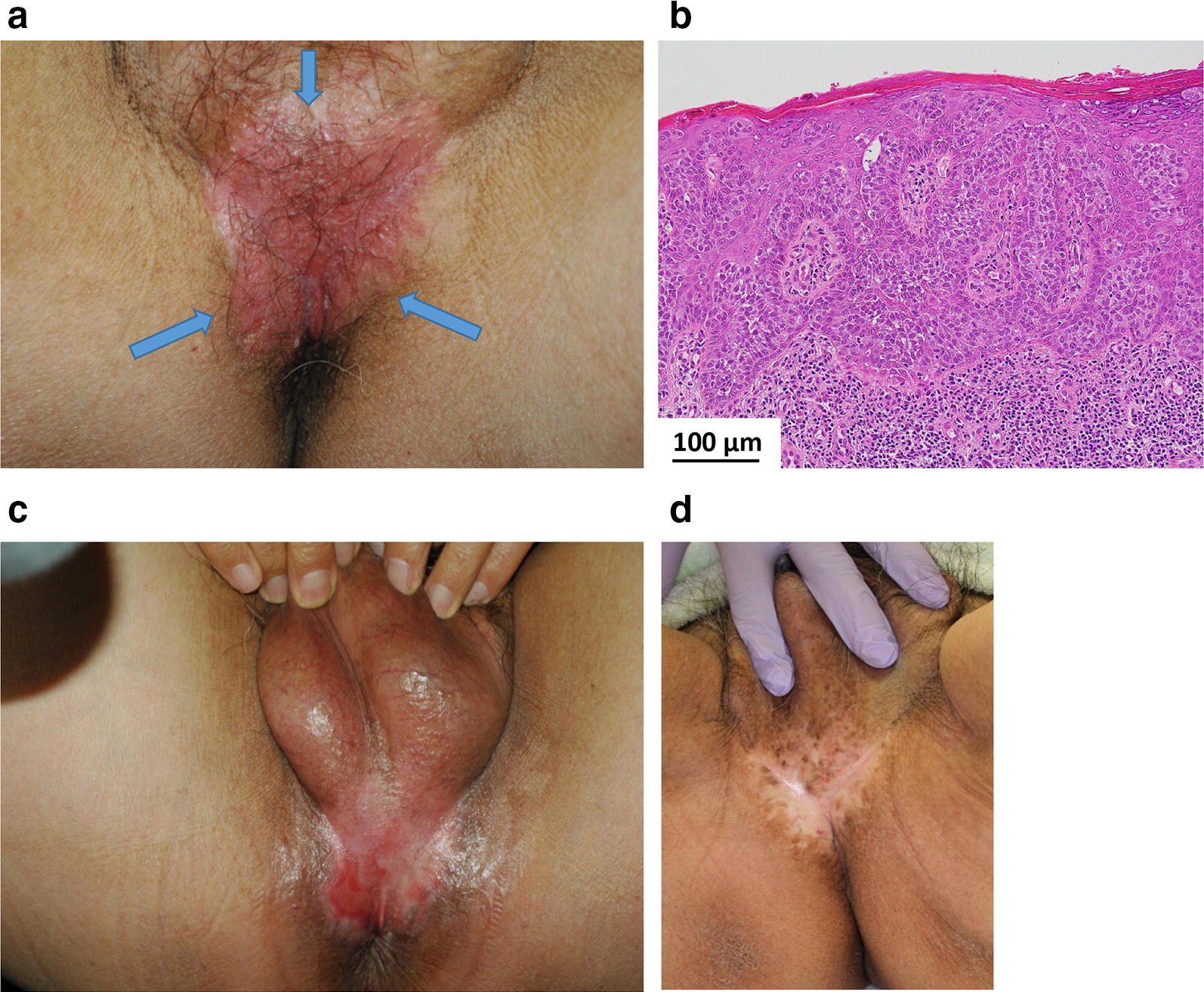
Macroscopic and microscopic images of a 73-year-old man with extramammary Paget’s disease. **a** External appearance before boron neutron capture therapy (BNCT): a pruritic, painful and persistent erythematous lesion (arrows) was on the perianal region and scrotum. **b** Microscopic findings: the histopathologic findings showed large, ovoid, infiltrating malignant epithelial cells with abundant cytoplasm, round nuclei and prominent nucleoli. **c** External appearance after BNCT (early reaction): BNCT delivered an absorbed dose of 7.3 Gy-Eq to the normal skin and 18 Gy-Eq to the tumor. The most severe adverse event in normal skin was erosion during the first 2 months, which was subsequently resolved with skin medication. **d** External appearance after BNCT (late reaction): he had a complete response with depigmentation of the lesion, and was alive and well without evidence of recurrence or adverse effects for 6.5 years after BNCT

**Fig. 5:**
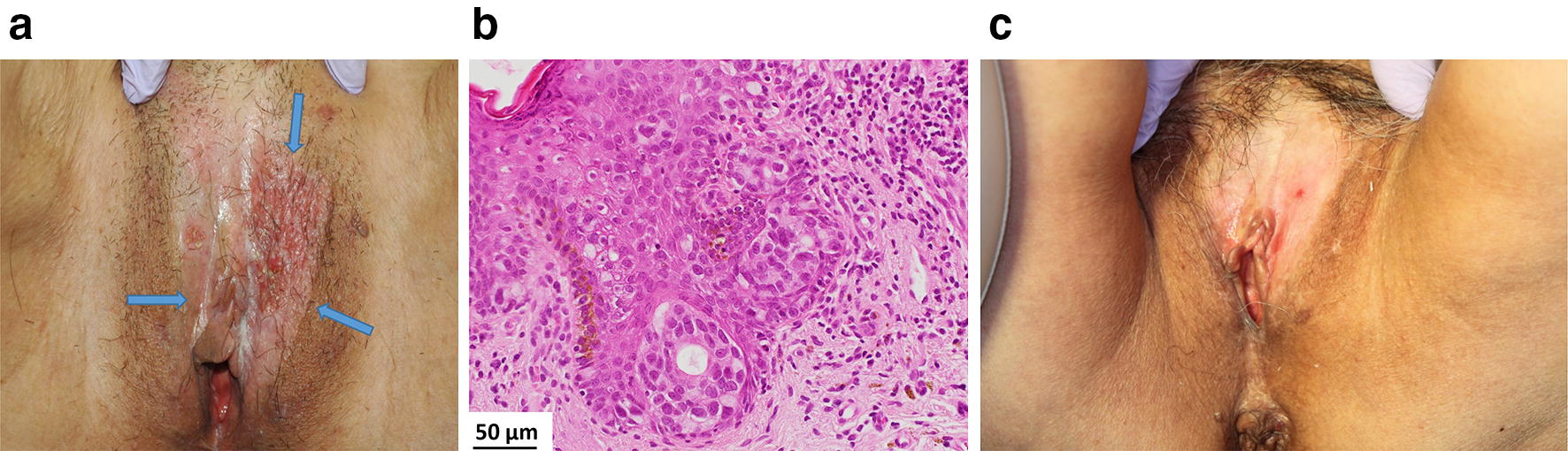
Macroscopic and pathologic images of a 69-year-old woman with extramammary Paget’s disease (EMPD). **a** External appearance before boron neutron capture therapy (BNCT): the labia and the vulva, presented as a pinkish, partially depigmented, and eczematous patch with irregular border in the mons pubic area (arrows). **b** Microscopic findings: pagetoid cells infiltrated all levels of the epidermis and slightly invaded the upper dermis. **c** External appearance after BNCT: absorbed doses following BNCT were 6.4 Gy-Eq to the normal skin and 20 Gy-Eq to the tumor. The EMPD lesion showed a complete response with depigmentation. She died of heart disease with no evidence of recurrence or adverse effects 3.2 years following BNCT

The three patients with EMPD showed similar responses in tumor and normal tissue after BNCT (Figs. 3, 4, 5). All patients achieved CR within 6 months and the most severe adverse event in normal tissue was moderate skin erosion during the first 2 months, which was subsequently resolved with a skin medication (Fig. 4c). Dysuria or contact pain persisted for 2 months and gradually diminished thereafter, and resolved completely within 4 months. One patient (Case 4) died of heart disease 3.2 years after treatment with no recurrence, whereas the remaining two patients were still alive and without evidence of local or regional recurrences at 6.5 and 6.9 years after BNCT.

## Discussion

Because VM and EMPD are very rare, no prospective, randomized, clinical trials have been performed to determine the effectiveness of various treatment options for these malignancies. At present, the most commonly recommended treatment is wide local excision of the lesion. However, this approach can be highly mutilating and can significantly diminish the quality of life. Effective alternative treatments for the primary tumor are therefore needed. High LET carbon ion radiotherapy is reportedly efficacious for cutaneous melanomas, with actual local control rates at 1 and 3 years of 85.7% and 42.9%, respectively [13]. However, no patients with EMPD and VM were included in this study [13]. Karasawa et al. [34] reported the outcomes of 23 patients with gynecologic melanomas treated with carbon ion radiotherapy. Fourteen of the melanomas were located in the vagina, 6 in the vulva, and 3 in the cervix. Total doses of 57.6 Gy-Eq in 16 fractions each were administered to 22 patients, and 64 Gy-Eq to one patient. Six patients showed complete responses, and partial responses were noted in 17 patients. Finally, the 3-year local control and overall survival rates were 49.9% and 53%, respectively; the authors concluded that carbon ion radiotherapy could be an acceptable alternative to surgery.

Surgery for EMPD of the genital region is limited from the prognostic point of view because of its multifocal nature and its frequent association with severe morbidity and functional impairment. Photon radiotherapy has been used in certain conditions, such as elderly patients who are medically unfit for surgery, or patients who refuse surgery, or as an alternative therapy for those with recurrence after repeated operations. Although optimal radiation doses have not been definitively determined, several authors [35, 36] have recommended 40–60 Gy. The morbidity associated with radiotherapy is minimal.

BNCT has three significant advantages over carbon ion therapy or conventional photon radiotherapy. *First*, although melanomas are generally considered to be resistant to conventional photon irradiation, they can be eradicated by BNCT, and both oxic and anoxic tumor cells are equally susceptible. *Second*, BNCT can be administered to relatively large areas, thereby allowing a wide margin, because the BPA selectively accumulates in both melanotic and non-melanotic tumor cells. These cells then are killed by the ^10^B(n,α)^7^Li capture reaction without significant damage to the surrounding normal tissue. In contrast with carbon ion radiotherapy, which has a Bragg peak, the dose in BNCT is uniformly delivered within the target volume. This advantage of BNCT is particularly useful for treating EMPD because histological involvement characteristically extends beyond the grossly visible lesion [37]. EMPD is usually multifocal with sub-clinical extension, which sometimes hinders the establishment of precise limits with normal skin. It has been reported that the surgical margin is positive despite wide excision with, grossly, a 2 cm margin. Biopsy examination to judge the margin cannot guarantee surgical margin negativity because the extent of histologic involvement is greater than that of the gross lesion [37]. Therefore, avoiding local recurrence following both surgery and radiotherapy requires adding a wide safety margin to the visible area [36, 37]. *Third*, BNCT is administered in a single fraction and high radiation doses can be delivered selectively to cancer cells. Hypothetically, doses as high as 60–80 Gy-Eq can be delivered to the malignant cells after the uptake and retention of BPA-F within approximately 1 h following its intravenous administration. In comparison, such radiation doses are usually administered over 6–7 weeks when conventionally fractionated photon radiation is used. This advantage enables patients to quickly resume their normal activities.

Our results are preliminary, as the patient cohort was very small. Furthermore, we based our dose calculations on data obtained from previous studies on cutaneous melanomas [30]. That is, we did not measure the boron concentration in each tumor because no easy way to do so directly was available, other than taking biopsies, followed by neutron irradiation to determine it by means of prompt gamma emission [31]. Nevertheless, we believe that the responses observed in tumor, skin, and mucosa following BNCT indicate that our calculations were accurate. Early and late responses, such as ulceration, necrosis, or residual tumor, did not occur during the 1.1–6.9 years of follow-up of the four patients. The most important goal of cancer treatment is to achieve tumor control while sparing surrounding normal tissue and preserve its function, and this was achieved in our patients with genital cancers.

Recent advances in immunotherapeutic approaches [38, 39] to treat metastatic melanoma combined with BNCT of the primary tumor might represent a breakthrough in treating this malignancy, which has a high propensity to metastasize. A Phase III clinical trial has shown that high-dose interferon-alpha (IFNα) can significantly increase overall survival [38]. A recent Phase III clinical trial also showed that cytotoxic T lymphocyte antigen-4 (CTLA-4) blockade with ipilimumab significantly improved overall survival [36]. Finally, targeting the programmed cell-death-1(PD-1) ligand with multiple anti-PD-1 monoclonal antibodies has been evaluated in Phase III trials and these also had impressive results [39]. Since BNCT spares normal cells, and more specifically immune effector cells, it may fit in ideally with immunotherapeutic approaches to treat VM. VM has an unfavorable prognosis and a relatively unpredictable biologic behavior and it tends to recur locally, and metastasize by hematogenous dissemination [3]. Thus, local BNCT and systemic immunotherapy are mutually complementary and potentially synergistic, because BNCT spares immune effector cells at the site of the tumor.

## Conclusions

This is the first clinical report of treatment of patients with VM and EMPD by BNCT, which resulted in complete local tumor control. Our results suggest that BNCT is a promising treatment modality for VM and EMPD, which were heretofore considered to be radio- and chemotherapeutically resistant.

## Abbreviations

BNCT: boron neutron capture therapy
BPA: para-boronophenylalanine
BPA-F: para-boronophenylalanine-fructose complex
CBE: compound biological effectiveness
CR: complete regression
EMPD: extramammary Paget’s disease
KUR: Kyoto University Research Reactor
LET: linear energy transfer
RBE: relative biological effectiveness
VM: vulvar melanoma
